# Next-generation protein sequencing and individual ion mass spectrometry enable complementary analysis of interleukin-6

**DOI:** 10.1007/s00216-025-06120-7

**Published:** 2025-10-01

**Authors:** Kenneth A. Skinner, Troy D. Fisher, Andrew Lee, Taojunfeng Su, Eleonora Forte, Aniel Sanchez, Michael A. Caldwell, Neil L. Kelleher

**Affiliations:** 1 Quantum-Si Incorporated, Branford, CT USA; 2 Proteomics Center of Excellence, Chemistry of Life Processes Institute, Northwestern University, Evanston, IL USA; 3 Department of Chemical and Biological Engineering, Northwestern University, Evanston, IL USA; 4 Department of Molecular Biosciences, Northwestern University, Evanston, IL USA; 5 Division of Nephrology, Department of Medicine, University of Illinois College of Medicine, Chicago, IL USA; 6 Division of Hematology Oncology, Department of Medicine, Feinberg School of Medicine, Northwestern University, Chicago, IL USA; 7 Department of Chemistry, Northwestern University, Evanston, IL USA

**Keywords:** Single-molecule protein sequencing, Next-generation protein sequencing, Platinum, Top-down mass spectrometry, Individual ion mass spectrometry, Proteoform

## Abstract

The vast complexity of the proteome currently overwhelms any single analytical technology in capturing the full spectrum of proteoform diversity. In this study, we evaluated the complementarity of two cutting-edge proteomic technologies—single-molecule protein sequencing and individual ion mass spectrometry—for analyzing recombinant human IL-6 (rhIL-6) at the amino acid, peptide, and intact proteoform levels. For single-molecule protein sequencing, we employed the recently released Platinum instrument. Next-generation protein sequencing (NGPS) on Platinum utilizes cycles of N-terminal amino acid recognizer binding and aminopeptidase cleavage to enable parallelized sequencing of single peptide molecules. We found that NGPS produces single amino acid coverage of multiple key regions of IL-6, including two peptides within helices A and C, which harbor residues that reportedly impact IL-6 function. For top-down proteoform evaluation, we used individual ion mass spectrometry (I^2^MS), a highly parallelized Orbitrap-based charge detection MS platform. Single ion detection of gas-phase fragmentation products (I^2^MS^2^) gives significant sequence coverage in key regions of IL-6, including two regions within helices B and D that are involved in IL-6 signaling. Together, these complementary technologies delivered a combined 52% sequence coverage, offering a more complete view of IL-6 structural and functional diversity than either technology alone. This study highlights the complementarity of these protein detection methods to cover protein segments relevant to biological interactions more comprehensively.

**Graphical Abstract:**

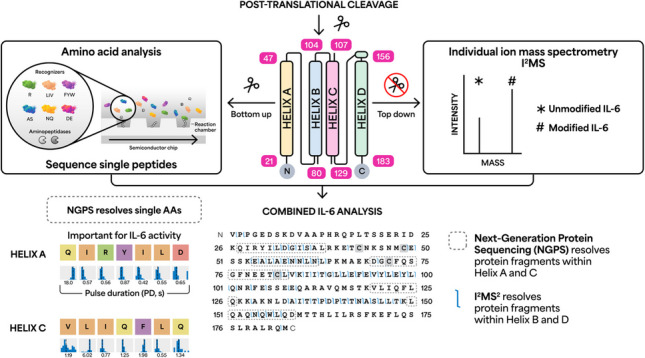

**Supplementary Information:**

The online version contains supplementary material available at 10.1007/s00216-025-06120-7.

## Introduction

Proteins are fundamental to nearly all biological processes, serving as key cellular components and prime drug targets [1, 2]. Protein-based therapies are among the top-selling pharmaceutical products [3]. The structure and function of proteins are determined by their primary amino acid sequence and post-translational modifications (PTMs), making the analysis of these features crucial for drug development and biopharmaceuticals.

Cytokines, such as interleukin-6 (IL-6), play a vital role in immune regulation, and engineered variants are widely used in therapeutic applications [4, 5]. In vivo, these essential proteins exist in multiple forms, or proteoforms [6], generated not only by genetic variation but also by alternative splicing [7], proteolysis [8], and various post-translational modifications (PTMs) [9, 10], all of which can affect their stability and interactions with biological targets. Unexpected amino acid substitutions, errors in protein synthesis, and variations in expression systems can give rise to a wide array of proteoforms of a recombinant protein with direct impact on potency and function [11]. Understanding and controlling these factors is essential for refining recombinant IL-6 formulations and optimizing their clinical efficacy.

Current approaches for therapeutic protein characterization, including intact mass analysis and multi-attribute methods (MAMs), have been deployed to great effect to support the growth of protein therapeutics in recent years. However, these approaches often do not completely characterize proteoforms, require significant expertise and overhead, and have limitations in sensitivity, mass range, and sample complexity [12, 13]—leading many to rely on western blot or other dated but accessible measures for characterization of expressed proteins. Several new technologies offer promising approaches to overcoming these challenges. Next-generation protein sequencing (NGPS) and individual ion mass spectrometry (I^2^MS) are highly parallelized, single-molecule approaches to analyze protein sequences and PTMs. NGPS uses engineered protein-based recognizers to sequence peptides with single amino acid resolution, while I^2^MS is a mass spectrometry technique that directly measures the intact mass of individual proteoform ions.

NGPS on the Platinum® and Platinum® Pro instruments marketed by Quantum-Si, Inc. employs fluorophore-labeled N-terminal amino acid (NAA) recognizers that reversibly bind cognate NAAs and aminopeptidases that reveal sequential NAAs to provide sequence and PTM information on immobilized Lys-C digested peptides [14]. In contrast to other recognizer-based methods, NGPS NAA-recognizer interactions produce multi-faceted kinetic signatures sensitive to adjacent amino acids and post-translational modifications, providing detailed sequence and PTM information about specific regions and enabling inferred protein-level identifications [14]. These capabilities are similar to conventional bottom-up proteomic workflows. However, Platinum aims to make detailed sequence characterization and protein identification available outside of traditional proteomics settings. Platinum is smaller and more affordable than commercially available mass spectrometers and does not require specialized lab space or expertise to analyze samples using kits and a cloud-based analysis suite available from Quantum-Si [15].

I^2^MS uses multiplexed Orbitrap-based charge detection mass spectrometry (CDMS) to directly measure the mass of individual proteoform ions, producing significantly simplified mass-domain spectra for mixtures of proteoforms without the challenges of deconvolution. The application of I^2^MS provides a > 500 × improvement in analytical sensitivity, > 10 × increase in mass range, and > 10 × higher resolution for the characterization of proteoforms over traditional intact mass and top-down mass spectrometry (TD-MS) approaches [16, 17]. These improvements enable input proteins to be larger, more dilute, and have more complex proteoform landscapes. Further, I^2^MS is compatible with automation, enabling automated sample processing and analysis [18]. Tandem-MS with the detection of individual ions (I^2^MS^2^) resolves fragment ions from the measured molecular ion to provide sequence analysis across the whole protein, confident protein identification, and localization of PTMs [16]. A version of this technology is marketed by Thermo Fisher Scientific as Direct Mass Technology mode on select MS instruments.

NGPS and I^2^MS utilize distinct fluorescence- or mass spectrometry–based techniques to characterize proteins and their proteoforms. In the context of biopharmaceutical quality control, there is an increasing demand for orthogonal methods—those that employ different physical principles to measure the same property—and for complementary approaches that provide a more comprehensive assessment of protein characteristics. Both I^2^MS and NGPS represent promising single-molecule technologies that meet these needs [15, 17]. Moreover, when used together, NGPS and I^2^MS can provide complementary insights, covering overlapping and distinct protein regions, thus enhancing overall sequence coverage and depth.

In this study, we used two independent technologies to analyze a recombinant human IL-6 (rhIL-6) sample: NGPS for single-molecule protein sequencing [14] and I^2^MS^2^ for intact mass profiling of proteoforms and readout of top-down fragmentation spectra [17]. By integrating these orthogonal methods for the first time, we show how they complement each other to provide more comprehensive coverage of key regions crucial for IL-6 function and therapeutic potential.

## Materials and methods

### Peptide sequencing on platinum

Experiments were conducted in accordance with the reagent kits and protocols described at https://www.quantum-si.com/resources/access/ [15]. Briefly, an aliquot of a stock solution of rhIL-6 sourced from AcroBiosystems (IL6-H4218) was diluted to a concentration of 1 µM in 100 µL Optima™ LC/MS grade water (Fisher Scientific, W64), reduced with TCEP, and cysteine residues were alkylated with chloroacetamide. Digestion of rhIL-6 with Lys-C was performed to generate peptides with C-terminal lysine residues. These peptides underwent diazotransfer and bioconjugation reactions to attach a macromolecular linker to the C-terminal lysines. After fluorescent quantification using included “Quant buffer,” conjugated peptides were immobilized in nanoscale reaction chambers on a semiconductor chip using the provided Loading solution. The chip was loaded into the Platinum instrument where loading quality control checks were performed. The sequencing mixture, consisting of aminopeptidases and six NAA recognizers that target 13 NAAs (R, L, I, V, F, Y, W, A, S, N, Q, D, E), was then added to the chip, and the sequencing run was initiated using the Platinum’s on-screen controls. During on-chip sequencing, fluorophore-labeled NAA recognizers reversibly bind cognate NAAs, producing recognition segments (RSs) and fluorescence properties that are captured by the semiconductor chip. To carry out the sequencing process, aminopeptidases cleave the peptide bond and expose the subsequent NAA for recognition [14]. This workflow was performed in duplicate for rhIL-6 with representative results included below.

### Analysis of sequencing data; analysis versions

Data analysis was performed using Quantum-Si’s cloud-based Platinum Analysis Software platform. Primary Analysis v2.5.1, Peptide Alignment v2.3.0, and Protein Inference v2.5.2 workflows were used. Details can be found in the Platinum Analysis Software Data Sheet (February 2, 2024). The Primary Analysis workflow is the first step in processing data, which characterizes the apertures across the chip based on peptide loading, recognizer activity, recognizer reads, and recognizer read lengths. These results are used as input for downstream analysis workflows.

### Peptide alignment v2.3.0 workflow

For the Peptide Alignment workflow, a reference sequence is required to call amino acids from the recognizer reads. Reads from the sequencing data were aligned to the FASTA reference of mature rhIL-6. Peptides were aligned based on the correspondence of observed recognition segments to the expected reference profile, using recognizer identity.

Peptide Alignment workflow also computes a false discovery rate (FDR) for each aligned peptide. This calculation is adapted from methods used in peptide identification by mass spectrometry [19], based on decoy peptide matching. Thus, FDR represents the relative number of alignments to the reference peptide sequence versus the total number of off-target alignments, Such as scrambled sequences with the same length as the target peptide. The standard cutoff for FDR is 0.1, or 10%.

### Protein inference v2.5.2 workflow

A pre-defined reference set of 8076 human proteins was used to infer proteins from unknown samples and confirm the identity of samples. The proteins in this reference panel span 10–70 kDa and contain at least three in silico Lys-C-digested peptides with three unique, visible residues. Inferred proteins are ranked by their respective Inference Score. The Inference Score is a natural log calculation of the FDR associated with the inferred protein.

### Sample preparation and data collection for I^2^MS and I^2^MS^2^ analysis

RhIL-6 (AcroBiosystems, IL6-H4218) was reconstituted in Optima™ LC/MS grade water (Fisher Scientific, W64), aliquoted, and stored at −80 °C according to the manufacturer’s recommendations until use. Samples were analyzed with the SampleStream Platform (Integrated Protein Technologies, Evanston, IL) [20] coupled to a modified Q-Exactive HF (Thermo Fisher Scientific; Bremen, Germany) mass spectrometer. Briefly, rhIL-6 was diluted to ~ 0.8 µM with water and transferred to a low-retention autosampler vial (Waters, 186009186). For each injection, 10 µL was buffer exchanged with SampleStream into 80 µL denaturing buffer (70:30 water:acetonitrile with 0.2% formic acid), deposited into a clean vial to mix, and then 75 µL was aspirated to infuse at ~ 0.1 µM. Relevant SampleStream parameters included a 125-µL focus volume, 225 µL/min focus flow rate, 60 °C flow cell temperature, and a 5-kDa molecular weight cutoff membrane. Source conditions included a custom nano-electrospray emitter (CoAnn Tech, TIP36007540-10), 1.8 kV spray voltage, 1.0 µL/min flow rate, and 320 °C inlet capillary temperature. Instrument parameters included RF 50%, 5e6 fixed AGC target, 120,000 resolution (at 200 *m/z*), 1 µscan, −1 kV central electrode voltage, 0.3 (arb) trapping gas pressure setting, 600–2500 *m/z* scan range, and a 78-min acquisition length (~ 4583 scans). The injection time for each acquisition was determined via automated ion control (AIC) to remain on the individual ion level [21].

For I^2^MS^2^ experiments, a charge state for each precursor was selected using The Fisher, a tool developed internally by the Kelleher group [22]. Briefly, ions within a 0.8 *m/z* isolation window width and 20 Da mass window width centered on a precursor mass were counted as ions corresponding to either the desired precursor or other species within an I^2^MS data file. The charge state that maximized the number of desired precursor ions and minimized the number of ions from different species was selected for isolation. Precursors were isolated and fragmented via higher energy collisional dissociation (HCD) normalized to charge state. Fragment ions were measured within a 150–2500 *m/z* scan range. Normalized collisional energy (NCE) and injection time were set manually to optimize fragmentation and ion counts. Fragmentation experiments were performed in duplicate with representative results included below.

### I^2^MS and I^2^MS^2^data processing and analysis

The I^2^MS charge assignment and ion mass determination was performed as previously described [17, 23].

A fragment search against the precursor amino acid sequence and expected PTMs was carried out using TDValidator (Proteinaceous, Inc., Evanston, IL). Fragment ions were identified by matching their isotopic distributions to theoretical isotopic distributions generated using an averagine model [24] and the Mercury7 [25] algorithm. To make ions searchable in TDValidator, neutral mass I^2^MS spectra were transformed into theoretical + 1 (M + H) distributions. All fragment ions were identified within a ± 10 ppm tolerance of their theoretical values for the isotopic distribution (max PPM tolerance) and ± 5 ppm tolerance for isotopologues within the same distribution (sub PPM tolerance). Other search metrics included a 1.5 S/N cutoff and 0.01 score cutoff. Spectra were manually curated to remove poor fragment ion matches. To calculate P-scores, fragment monoisotopic masses were generated with the THRASH algorithm in TDValidator using a 1.5 S/N cutoff, + 1 charge (+ 30 charge for *m/z* domain data), 30,000 Da maximum mass, and 0.9 minimum RL value and uploaded to ProSight Lite (http://prosightlite.northwestern.edu/) with ± 10 ppm tolerances.

## Results and discussion

**Fig. 1:**
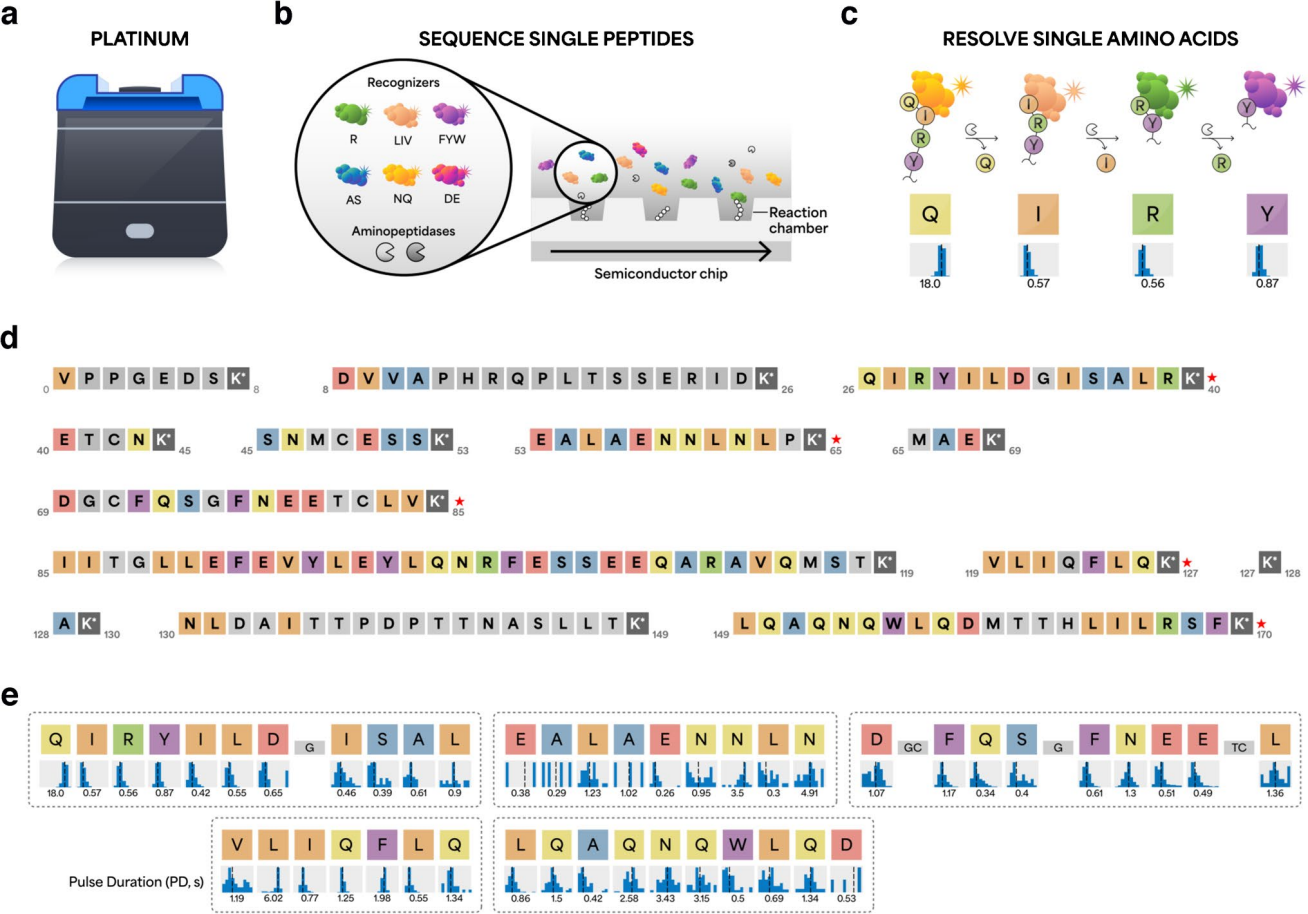
Next-generation protein sequencing (NGPS) of IL-6 with the Platinum instrument. **a** The Platinum instrument sequences single peptide molecules with single amino acid resolution. **b** Sequencing kits include semiconductor chips, aminopeptidases, and six dye-labeled NAA recognizers that reversibly bind 13 target NAAs. **c** Binding of dye-labeled NAA recognizers generates kinetic information indicating which amino acid is being detected. **d** Results of in silico Lys-C digestion of mature IL-6. Missing C-terminal residues 171–183 lack a lysine residue (K) and thus are not amenable for peptide capture and sequencing on Platinum. Colored boxes indicate potential recognition events. Gray boxes indicate amino acids not amenable to recognition. Red asterisks indicate automated alignments of sequencing data to reference sequence. **e** NGPS of rhIL-6 on Platinum detects five IL-6 peptides (dotted rectangles). NAA recognizers elicit kinetic signatures such as pulse duration (PD), which reflects the affinity between specific recognizers and NAAs. PD histograms represent the statistical distribution of kinetic data for all pulses associated with a specific residue and support inference of the corresponding amino acid. Values are reported as the median of the mean across reaction chambers

To evaluate the ability of each proteomic technology to detect key regions of rhIL-6, we first performed NGPS of rhIL-6 using the Platinum instrument (Fig. 1a). The Platinum platform includes three components: kits for bottom-up protein processing and sequencing; a benchtop Platinum instrument that accommodates semiconductor chips for sequencing of polypeptides (Fig. 1b and c); and cloud-based software for analysis of sequencing data (Fig. 1d and e).

For on-chip sequencing, surface-immobilized peptides are exposed to a mixture of freely diffusing NAA recognizers and aminopeptidases (Fig. 1b) [14]. Six NAA recognizers, labeled with different fluorophores, reversibly bind 13 target NAAs (Fig. 1b) and elicit characteristic pulsing patterns upon binding to each NAA (Fig. 1c). The semiconductor chip converts fluorescence signals into digital readouts, enabling real-time sequencing of single peptide molecules in parallel [14]. Cycles of binding and cleavage proceed to sequentially reveal the order of NAAs and enable identification of the peptide sequence [14].

For analysis of sequencing data, only peptides that meet specific thresholds (see “Materials and methods,” section “Peptide Alignment v2.3.0 workflow”) are eligible for high-confidence alignment to the reference rhIL-6 sequence (red asterisks, Fig. 1d). Based on these criteria, three rhIL-6 peptides (V_1_PPGEDSK_8_, E_41_TCNK_45_, and M_66_AEK_69_) are ineligible for alignment. In addition, the C-terminal segment 171–183 (E_171_FLQSSLRALRQM_183_) does not contain K and thus is not amenable to conjugation and on-chip sequencing. NGPS sequences five rhIL-6 peptides (Fig. 1e) and determines the identity of 46/183 single amino acids, indicating ~ 25% amino acid level coverage within rhIL-6 (Fig. 1e).

To support high-confidence identification of IL-6, we also examined the false discovery rate (FDR) for each peptide and used Protein Inference analysis (see “Materials and methods,” section “Protein Inference v2.5.2 workflow”) to determine the specificity of IL-6 mapping relative to a protein panel. Platinum analysis outputs FDR for each sequenced peptide using a target-decoy approach that is analogous to methodologies employed in MS, with a cutoff set at 0.1 (10%). FDR scores for each peptide are Q_27_IRYILDGISALRK_40_ (FDR: 0.0), V_120_LIQFLQK_127_ (FDR: 0.0), D_70_GCFQSGFNEETCLVK_85_ (FDR: 0.02), E_54_ALAENNLNLPK_65_ (FDR: 0.07), and L_150_QAQNQWLQDMTTHLILRSFK_170_ (FDR: 0.09). Interestingly, an FDR score of 0.0 was computed for peptide V_120_LIQFLQK_127_, despite contiguous isobaric residues leucine (L) in position 2 and isoleucine (I) in position 3 (Fig. 1e). The recognizer for branched chain NAAs (LIV) exhibits differential pulse duration (PD) profiles for L (6.02 s) and I (0.77 s), discerning the order of L and I residues with the same mass. This example demonstrates that a single NAA-recognizer can differentiate NAAs with similar physicochemical properties on the basis of differences in PD. In addition to FDR, we also used the Protein Inference analysis software, “Protein Inference v2.5.2 workflow” which screens the sequencing data against a reference panel of 8076 proteins. IL-6 was identified as the top protein hit with 99.99% confidence (see Electronic Supplementary Material Fig. S1). These results demonstrate NGPS confidently identifies rhIL-6 with single amino acid resolution.

**Fig. 2:**
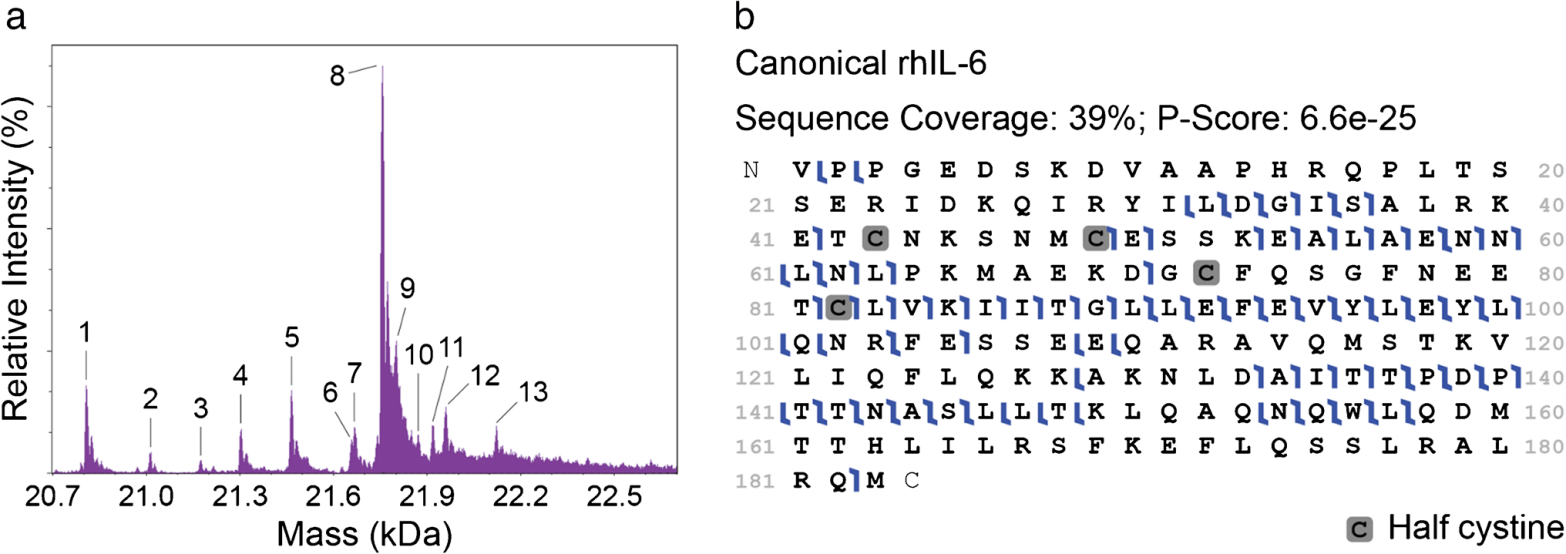
I^2^MS detects intact rhIL-6 proteoforms, and I^2^MS^2^ covers broad regions of rhIL-6 primary structure. **a** The application of I^2^MS to TD-MS enables measurement of the intact mass of intact protein ions of canonical rhIL-6 (~ 20.8 kDa) labeled 1. Higher mass proteoforms (PFRs) labeled 2–13 indicate the addition of PTMs. **b** TD-MS provides broad sequence coverage of canonical rhIL-6 (PFR 1) by higher energy collision dissociation (HCD), achieving 39% sequence coverage. Shaded half cystines indicate cysteine residues involved in disulfide bonds

Next, we deployed the same rhIL-6 sample for intact mass measurement via I^2^MS. I^2^MS accurately identifies unmodified rhIL-6 (~ 20.8 kDa) and several higher molecular weight proteoforms up to ~ 22 kDa (Fig. 2a), consistent with reports from the manufacturer and others that glycosylation is expected for our HEK293-expressed rhIL-6 [26]. For comparison, a conventional composite *m/*z domain spectrum is provided in Electronic Supplementary Material Fig. S2. Human IL-6 has been shown to undergo a number of PTMs, including O- and N-linked glycosylation and phosphorylation, though few reports detail the composition or localization of IL-6 O-glycans [9, 27]. Using the intact masses of these proteoforms, the composition of the putative O-glycans was determined by searching the GlyGen database [28] for those glycans corresponding to the observed intact mass shift, and their chemical formula and mass were calculated using the publicly available NIST Glyco Mass Calculator [29] (see Electronic Supplementary Material Table S1). Thus, I^2^MS enables the observation of intact proteins and proteoforms, which is distinct from the data produced by NGPS.

We then isolated and fragmented discrete rhIL-6 proteoforms via I^2^MS^2^. Using higher energy collision dissociation (HCD), I^2^MS^2^ achieves 39% fragmentation coverage of the unmodified form of rhIL-6, with shared and distinct regions identified by NGPS (Figs. 1e and 2b). Of the 12 specified higher mass species, 7 of the more abundant species showed satisfactory ion counts of the desired precursor with minimal co-isolation of other species (PFRs 4, 5, 8, 9, 11, 12, 13) using The Fisher, an in-house tool for determining I^2^MS^2^ isolation windows from the intact I^2^MS^2^ spectrum. I^2^MS^2^ results of these selected higher mass species are consistent with putative O-glycans with compositions of N-acetyl hexosamine, hexose, deoxyhexose, and sialic acid variably located towards the C-terminus in Helix D and the adjacent C-D loop. Localization of the O-glycans at specific serine or threonine residues along the base rhIL-6 sequence was predicted using the OGP repository [30]. Sites T6, T48, S49, T117, T147, T166, T170, T171, and T177 generated a probability for O-glycosylation greater than 0.5 and thus were used as potential O-glycan locations. Canonical rhIL-6 and selected higher mass species with their respective O-glycan composition and localization are Supported with fragment map P-scores ranging from 6.6E^−25^ to 2.3E^−04^ (see Electronic Supplementary Material Fig. S3-S10 and Online Resource 1). The O-glycan localization (T147, T166, and T177 across all glycoforms) which generated the smallest P-score for each proteoform is reported. O-glycosylation located towards the C-terminus of human IL-6 is consistent with that reported in lung adenocarcinoma cells isolated from malignant pleural effusion [27], thus demonstrating the high utility of I^2^MS and I^2^MS^2^ for measuring specific glycosylated proteoforms of IL-6 associated with disease.

Both NGPS and I^2^MS^2^ detect the E_54_ALAENNLNL_63_ sequence region (Figs. 1e and 2b). Also, while NGPS provides single amino acid resolution of the D_70_GCFQSGFNE_79_ fragment, only a single residue cleavage was observed within this peptide by I^2^MS^2^ (Figs. 1e and 2b). Similarly, NGPS reports the sequence of the V_120_LIQFLQK_127_ peptide (Fig. 1e), which also contains no residue cleavages by I^2^MS^2^ (Fig. 2b).

For the region encompassing Q_27_IRYILDGISAL_38_ (Figs. 1e and 2b), NGPS and I^2^MS^2^ provide complementary coverage. While NGPS enables single amino acid resolution of 11/12 amino acids (Fig. 1e), I^2^MS^2^ detects fragment ions on either side of the glycine (G) residue, which is not detected by NGPS due to the lack of a G recognizer (Fig. 1b). While NGPS does not currently provide a C recognizer, I^2^MS^2^ detects C-containing fragments C_43_NKSNMCESSK_53_ and C_82_LVKIITGLLEFEVYLEYLQNR_103_ (Fig. 2b). In addition to this 22-amino-acid-long internal fragment, I^2^MS^2^ also provides near complete amino acid coverage across N_131_LDAITTPDPTTNASLLTK_149_ (Fig. 2b). Both NGPS and I^2^MS^2^ sequence the C-terminal region of rhIL-6 encompassing L_150_QAQNQW_156_ (Figs. 1e and 2b), which contains the only tryptophan (W) in IL-6 [31, 32]. Interestingly, amino acids within this fragment have been implicated in IL-6 binding interactions with receptors.

**Fig. 3:**
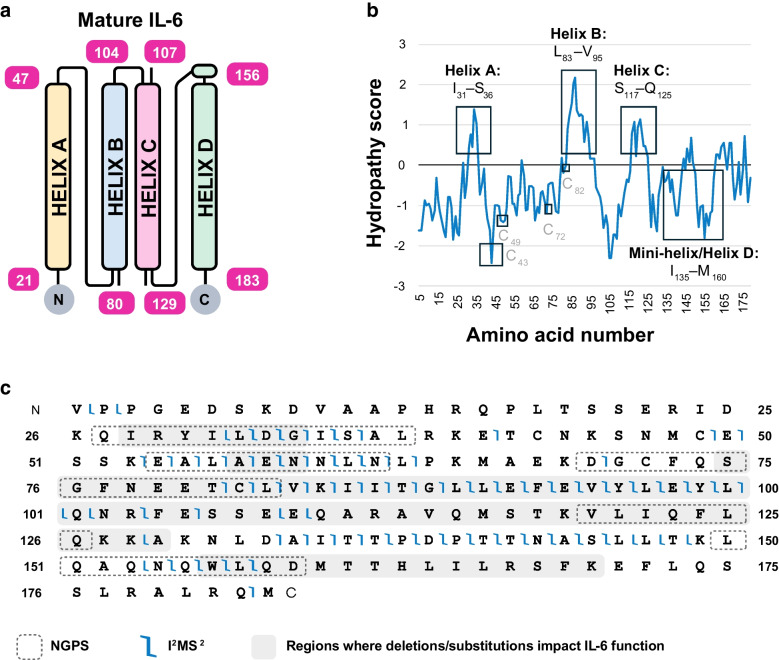
NGPS and I^2^MS/I^2^MS^2^ cover hydrophobic and hydrophilic regions of IL-6 important for IL-6 interactions. **a** Mature human IL-6 schematic. Numbering is approximate based on a reported three-dimensional structure of human IL-6. **b** Kyte-Doolittle hydropathy plot reports surface-exposed regions. Positive scores correspond to hydrophobic regions. Negative scores correspond to hydrophilic regions. Amino acid sequence position is on the *x*-axis. Average hydropathy score is calculated for windows of 9 amino acids. **c** NGPS (Platinum) and I^2^MS provide broad coverage of IL-6, combining to resolve 52% of single amino acids with coverage reported in key regions known to impact IL-6 interactions (shaded in gray)

Overall, our results demonstrate NGPS and TD-MS provide sequence information for overlapping and distinct regions within rhIL-6. To place these sequencing results in the context of IL-6 tertiary structure and function, we mapped the amino acid regions detected by NGPS (Fig. 1e) and I^2^MS^2^ (Fig. 2b) using an IL-6 crystal structure as a reference [33] (Fig. 3a).

IL-6, a four-helix bundle cytokine that is subject to differential signal peptide cleavage and glycosylation [8, 10, 34], is a multi-functional protein that transmits cellular signaling via IL-6 receptor alpha (IL-6R) and beta (gp130) [35]. The molecular information for IL-6 binding and activity is enabled via adoption of a four-helix fold, with a mini-helix before Helix D [33]. Each alpha helix contains 20–30 amino acids, with long AB and CD loops that accommodate an up-up-down-down helical orientation (Fig. 3a). Previous studies indicate that multiple segments within IL-6 topology are necessary for biological interactions [36]. Hence, broad coverage of IL-6 sequence is key for elucidating residues important for IL-6 interactions, which occur via hydrophobic and hydrophilic interactions spread across different domains.

To determine if NGPS and I^2^MS provide broad coverage of the helices and loop regions, we generated a Kyte-Doolittle hydropathy plot [37] (Fig. 3b). Interestingly, NGPS and I²MS analyze broad regions of IL-6 that are hydrophobic (positive scores) and hydrophilic (negative scores) (Fig. 3b). NGPS resolves single amino acids within peptide Q_27_IRYILDGISAL_38_ (Fig. 3c), located within Helix A, which is one of the most hydrophobic regions in IL-6 (Fig. 3b). Further, NGPS also detects V_120_LIQFLQK_127_ located within a hydrophobic portion of Helix C (Fig. 3b).

The most hydrophobic portion of IL-6 encompasses C_82_LVKIITGLLEFEVYLEYLQNR_103_ within Helix B (Fig. 3b), an internal fragment that I^2^MS detects (Fig. 3c). While this fragment is nonpolar, other cysteine-containing segments lie within hydrophilic regions (Fig. 3b). I^2^MS also discerns N_131_LDAITTPDPTTNASLLTK_149_ located within an amphipathic region of the CD loop and the mini-helix that precedes Helix D (Fig. 3a, c). Both I^2^MS and NGPS cover amino acids within Helix D (Fig. 3c), a C-terminal region that contains a stretch of amphipathic residues (Fig. 3b). Overall, NGPS and I^2^MS sequence various regions of IL-6 with different amino acid compositions and properties, highlighting the complementarity of these orthogonal analytical methods (Fig. 3c).

For sequence structure-function analysis, we performed a literature survey to determine which sequenced regions are relevant to IL-6 interactions. Removal of amino acids within the Q_27_IRYILD_33_ segment of Helix A reduces or abolishes IL-6 activity [36, 38] (Fig. 3c). Within the AB loop, E_54_ALAENNLNL_63_ contains residues important for IL-6 binding to IL-6R [39] (Fig. 3c). Similarly, D_70_GCFQSGFNE_79_ harbors residues such as phenylalanine (F), both of which are relevant for IL-6 binding interactions (Fig. 3c) [39–41]. We demonstrate that NGPS resolves both F residues within D_70_GCFQSGFNE_79_ (Fig. 1e, Fig. 3c). NGPS also detects peptide V_120_LIQFLQK_127_ within Helix C (Fig. 1e, Fig. 3c). Notably, a segment including V_120_LIQFLQK_127_ is truncated in an IL-6 splice variant with altered signaling (Fig. 3c) [7]. The mini-helix and amphipathic D helix contain a stretch of leucine and polar Q/N residues that influence IL-6 binding [31, 32]. Therefore, both NGPS and I^2^MS detect regions of IL-6 relevant to its biological function and interactions.

Limitations of this study include the use of recombinant IL-6 and, as a proof-of-concept study, its small sample size. Future studies will aim to expand the scope of analysis for combining NGPS and I^2^MS^2^ across a broader range of target proteins and proteoforms. Because both Platinum and I^2^MS do not utilize any online separations, managing sample complexity and dynamic range will be a key consideration for extension to other sample types such as cell lysates, media, or biofluids. Provided the initial steps can be optimized appropriately, we see a straightforward path for the application of both technologies to immunoprecipitates and SDS-PAGE gel bands. Indeed, applications of I^2^MS to complex immunoprecipitated samples and direct sampling have recently been demonstrated [22, 42].

## Conclusion

Taken together, our data demonstrate complementarity of single-molecule protein sequencing (NGPS on Platinum) and I^2^MS to cover key regions of IL-6. While NGPS provided single amino acid resolution for fragments like V_120_LIQFLQK_127_, which are essential for IL-6 receptor binding, I^2^MS enabled detection of larger proteoforms, providing critical information on PTMs, such as glycosylation, that affect IL-6’s stability and bioactivity. When combined, NGPS and I^2^MS cover many key regions of IL-6, achieving 52% combined sequence coverage of single amino acids. This sets up improvements in technology and informs future studies of how polymorphisms or mutations affect PTMs on endogenous IL-6. These complementary datasets underscore the potential of combining single-molecule protein sequencing and mass spectrometry to obtain a more comprehensive picture of IL-6 structural and functional diversity, which is vital for understanding its therapeutic potential.

## Supplementary Information

Below is the link to the electronic supplementary material.

ESM 1 (DOCX 8.50 MB)

ESM 2 (XLSX 94.7 KB)

## Data Availability

Raw and processed individual ion mass spectrometry data files and processed Platinum data files are available in the MassIVE data repository and can be accessed via accession MSV000098537.
