# Supplementary material for: Next-generation protein sequencing and individual ion mass spectrometry enable complementary analysis of interleukin-6
Source: Anal Bioanal Chem. 2025 Oct 1;417(28):6291–9. doi: 10.1007/s00216-025-06120-7 (PMC12598678; doi:10.1007/s00216-025-06120-7)
Supplement: Supplementary file 1 — (DOCX 8.50 MB) [file 216_2025_6120_MOESM1_ESM.docx]

**Supplementary Information**

Next-Generation Protein Sequencing and individual ion mass spectrometry enable complementary analysis of interleukin-6

Kenneth A. Skinner^1^; Troy D. Fisher^2^; Andrew Lee^3^; Taojunfeng Su^3^; Eleonora Forte^4^; Aniel Sanchez^2^; Michael A. Caldwell^2,5^; Neil L. Kelleher^2,3,5#^

^1^Quantum-Si Incorporated, Branford, Connecticut, USA

^2^Proteomics Center of Excellence, Chemistry of Life Processes Institute, Northwestern University, Evanston, Illinois, USA

^3^Departments of Molecular Biosciences, Chemistry and Chemical and Biological Engineering, Northwestern University, Evanston, IL, USA

^4^ Department of Medicine, Division of Nephrology, University of Illinois College of Medicine, Chicago, IL, USA

^5^Department of Medicine, Division of Hematology Oncology, Feinberg School of Medicine, Northwestern University, Chicago, IL, USA

#Corresponding author email: [n-kelleher@northwestern.edu](mailto:n-kelleher@northwestern.edu)


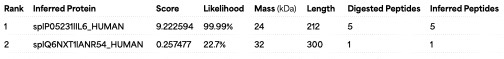


**Fig. S1 Protein inference v2.5.2 aligns observed kinetic signatures from sequencing data of rhIL-6 to a database of predicted kinetic signatures.** Protein Score is an estimate of the likelihood of protein identity. While ankyrin repeat domain-containing protein 54 (UniProt ID: Q6NXT1) contains peptide QIIHMLREYLERLGQHEQRERLDDLCTRLQMTSTK, which is similar in sequence to QIRYILDGISALRK in rhIL-6, Platinum unambiguously identifies rhIL-6.

**Table S1. Intact Proteoforms of rhIL-6 detected with I**^2^**MS.** 13 proteoforms of rhIL-6 were detected by I^2^MS and abundant species were targeted for tandem-MS with I^2^MS^2^. Based on the intact masses, putative glycan compositions were proposed and supported with I^2^MS^2^ data when available.

| PFR | Theoretical Monoisotopic Mass (Da) | Observed Monoisotopic Mass (Da) | Mass Error (ppm) | Half Cystine Sites | Putative O-Glycan Composition | Glycan Chemical Formula (unbound) | Monoisotopic Glycan Mass (Da) | I^2^MS^2^? |
| --- | --- | --- | --- | --- | --- | --- | --- | --- |
| 1 | 20795.58 | 20795.55 | -1.55 | C72, C78, C101, C111 | N/A | N/A | N/A | Yes |
| 2 | 20998.66 | 20998.64 | -1.03 | C72, C78, C101, C111 | HexNAc(1) | C8H15NO6 | +221.08994 | No |
| 3 | 21160.71 | 21160.69 | -1.16 | C72, C78, C101, C111 | HexNAc(1) Hex(1) | C14H25NO11 | +383.14276 | No |
| 4 | 21289.76 | 21289.72 | -1.74 | C72, C78, C101, C111 | HexNAc(1) NeuAc(1) | C19H32N2O14 | +512.18535 | Yes |
| 5 | 21451.81 | 21451.79 | -0.93 | C72, C78, C101, C111 | HexNAc(1) Hex(1) NeuAc(1) | C25H42N2O19 | +674.23818 | Yes |
| 6 | 21646.87 | 21646.84 | -1.52 | C72, C78, C101, C111 | HexNAc(1)  Hex(4) | C32H55NO26 | +869.30123 | No |
| 7 | 21654.89 | 21654.86 | -1.35 | C72, C78, C101, C111 | HexNAc(2) Hex(1) NeuAc(1) | C33H55N3O24 | +877.31755 | No |
| 8 | 21742.91 | 21742.90 | -0.25 | C72, C78, C101, C111 | HexNAc(1)  Hex(1)  NeuAc(2) | C36H59N3O27 | +965.33359 | Yes |
| 9 | 21785.95 | 21785.89 | -2.64 | C72, C78, C101, C111 | HexNAc(4)  Hex(1) | C38H64N4O26 | +992.38088 | Yes |
| 10 | 21858.99 | 21858.91 | -3.62 | C72, C78, C101, C111 | HexNAc(3)  dHex(2)  Hex(1) | C42H71N3O29 | +1081.41732 | No |
| 11 | 21905.98 | 21905.95 | -1.30 | C72, C78, C101, C111 | HexNAc(1)  dHex(2) Hex(2)  NeuAc(1) | C43H72N2O32 | +1128.40682 | Yes |
| 12 | 21947.01 | 21946.97 | -1.60 | C72, C78, C101, C111 | HexNAc(2)  dHex(2) Hex(1)  NeuAc(1) | C45H75N3O32 | +1169.43337 | Yes |
| 13 | 22109.06 | 22109.03 | -1.26 | C72, C78, C101, C111 | HexNAc(2)  dHex(2) Hex(2)  NeuAc(1) | C51H85N3O37 | +1331.48619 | Yes |

**
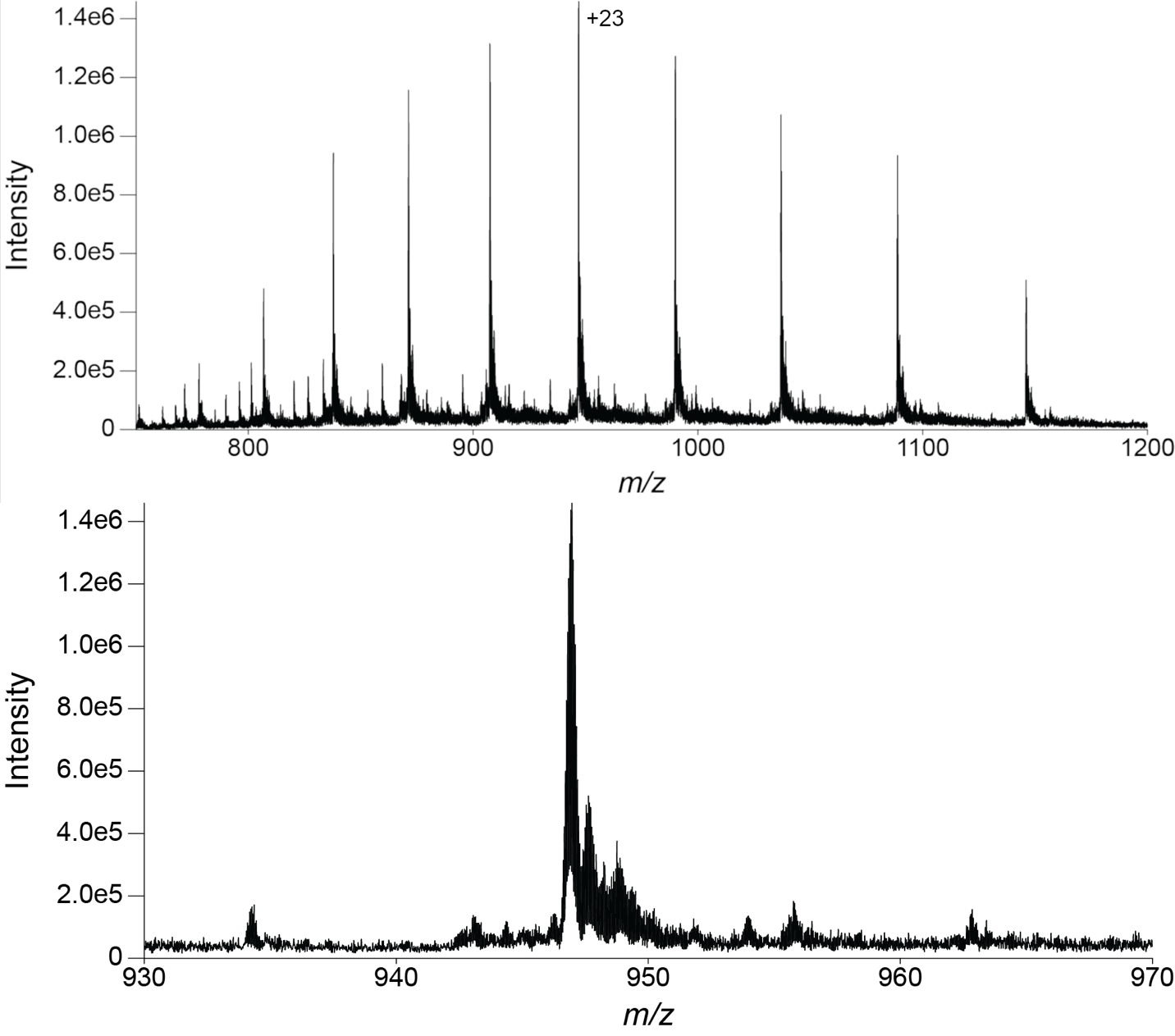
**

**Fig. S2** (Top) Composite *m/z* spectrum for the rhIL-6 proteoform landscape. (Bottom) Composite *m/z* spectrum showing presence of multiple rhIL-6 proteoforms of charge state +23.

**
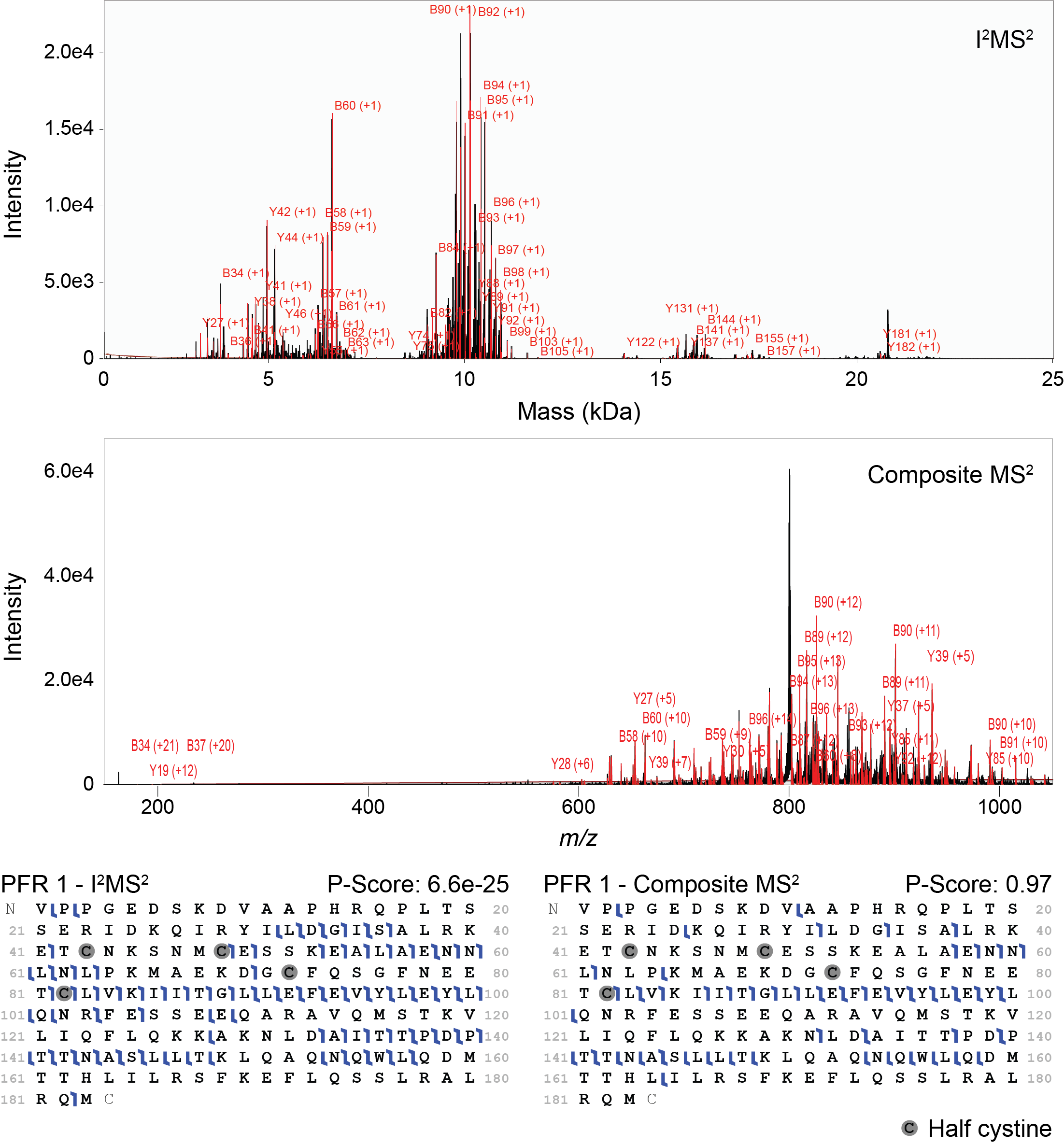
**

**Fig. S3** I^2^MS^2^ and composite MS^2^ spectra and the corresponding graphical fragment maps for rhIL-6 proteoform 1. Shaded half cystines indicate cysteine residues involved in disulfide bonds.

**
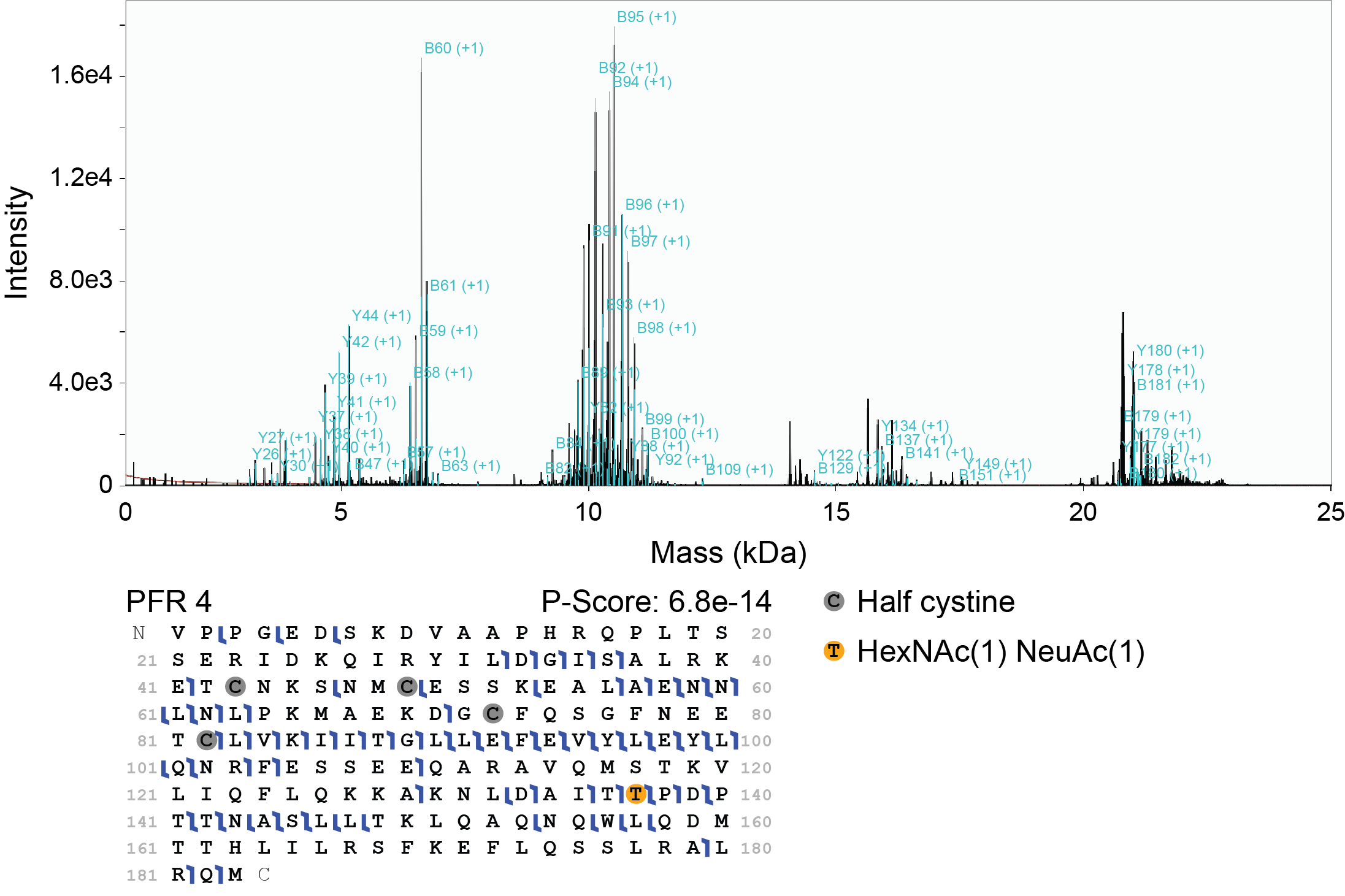
**

**Fig. S4** I^2^MS^2^ spectrum and graphical fragment map for rhIL-6 proteoform 4. Shaded half cystines indicate cysteine residues involved in disulfide bonds.

**
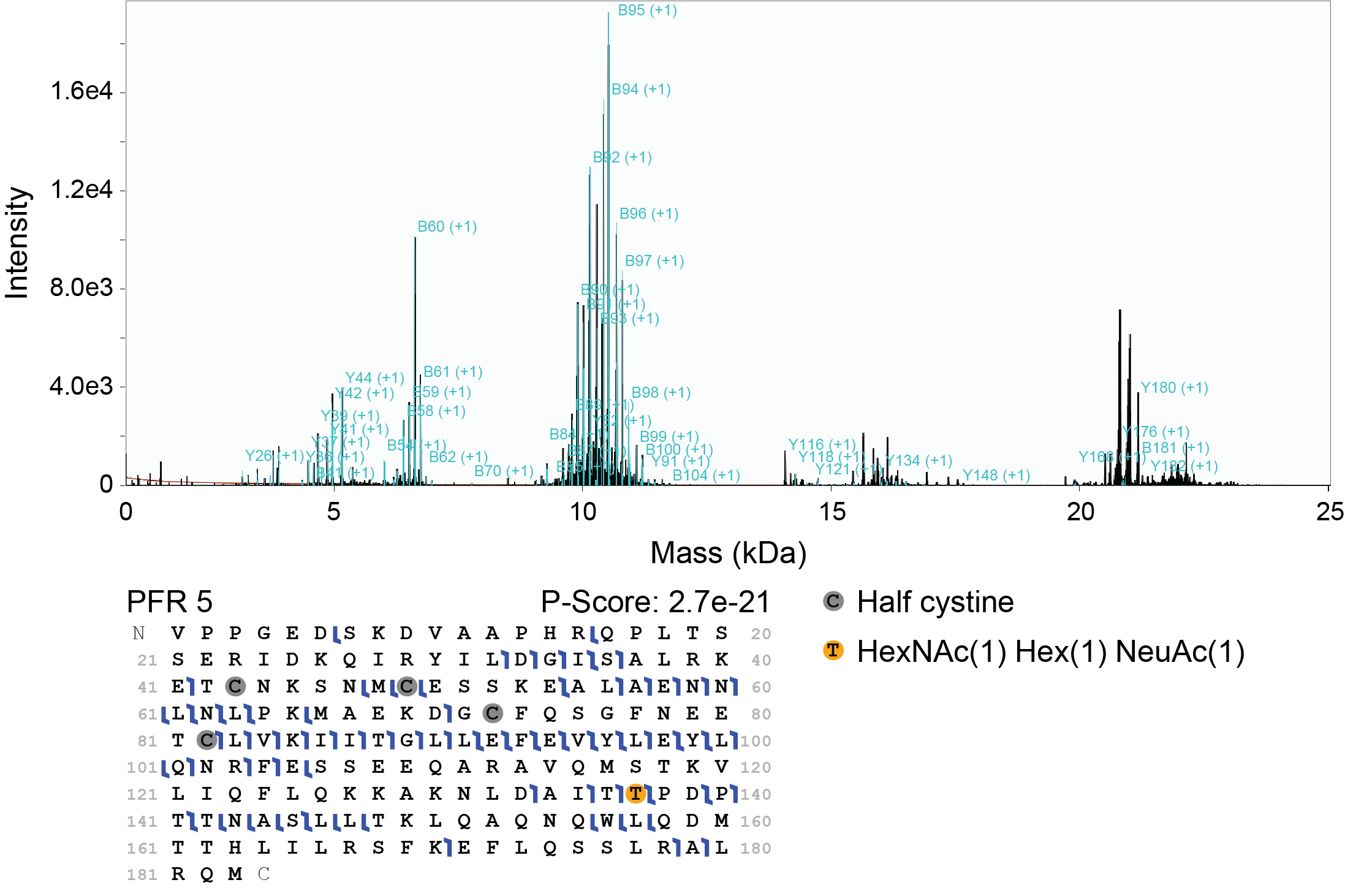
Fig. S5** I^2^MS^2^ spectrum and graphical fragment map for rhIL-6 proteoform 5. Shaded half cystines indicate cysteine residues involved in disulfide bonds.

**
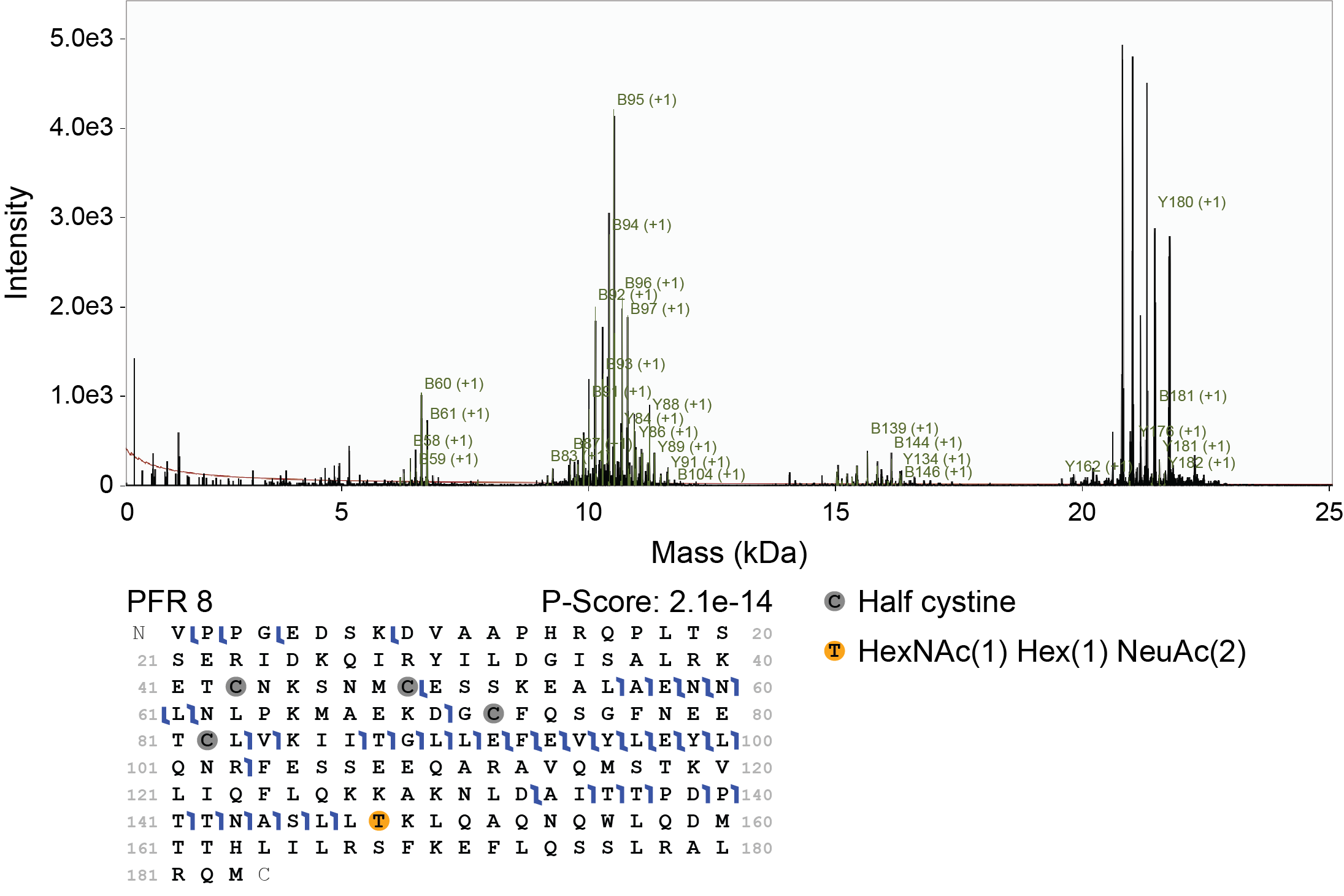
**

**Fig. S6** I^2^MS^2^ spectrum and graphical fragment map for rhIL-6 proteoform 8. Shaded half cystines indicate cysteine residues involved in disulfide bonds.


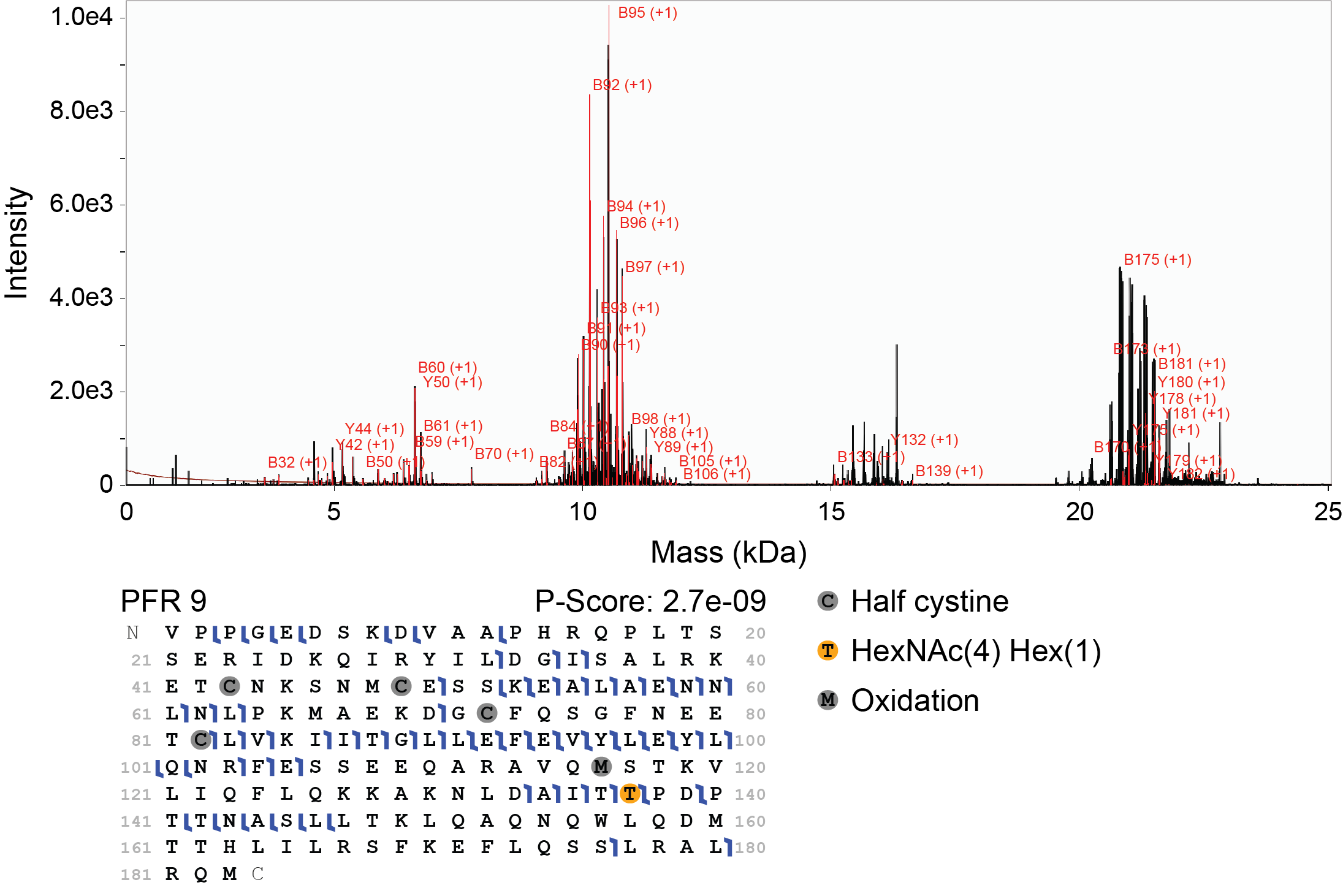


**Fig. S7** I^2^MS^2^ spectrum and graphical fragment map for rhIL-6 proteoform 9. Shaded half cystines indicate cysteine residues involved in disulfide bonds.

**
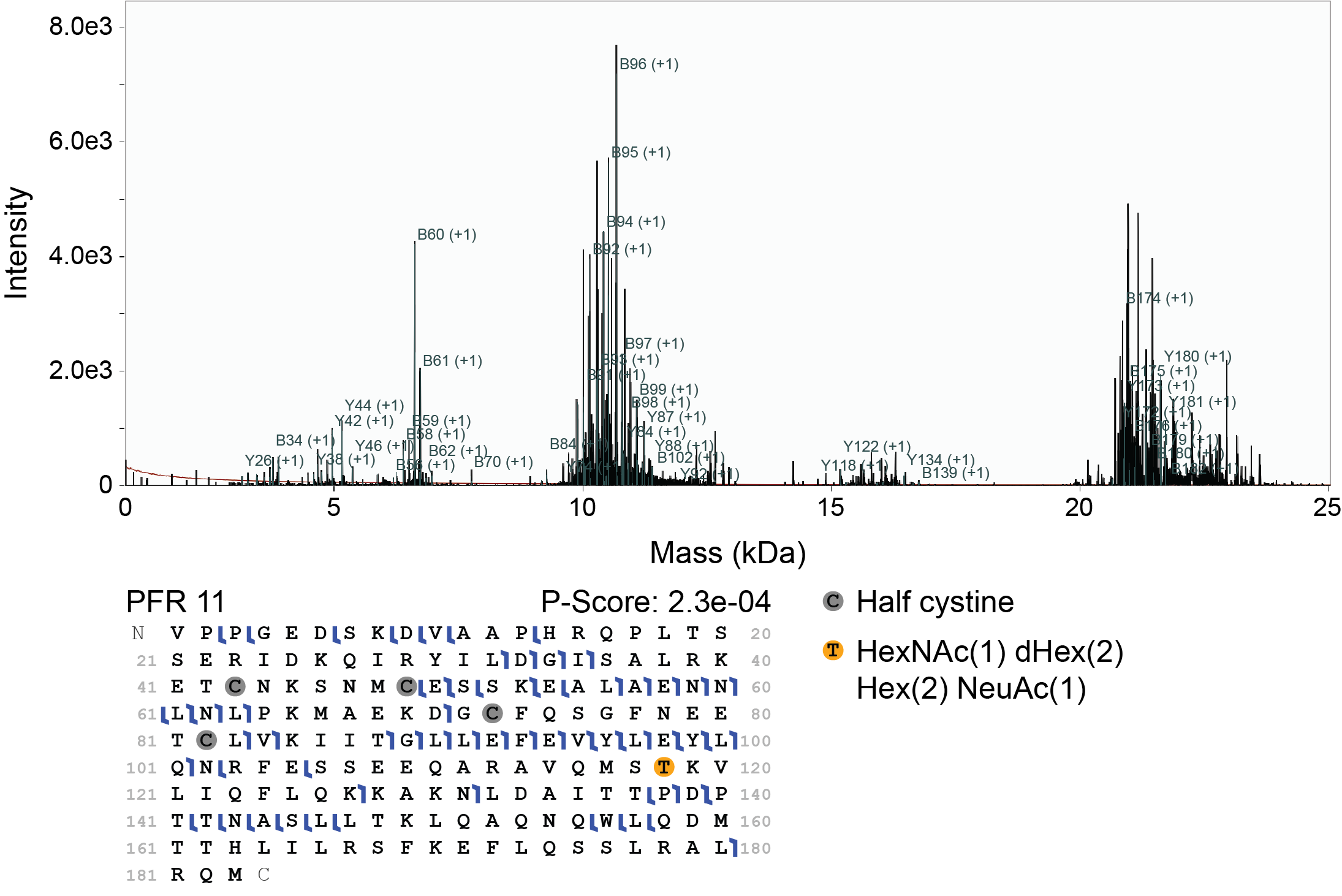
**

**Fig. S8** I^2^MS^2^ spectrum and graphical fragment map for rhIL-6 proteoform 11. Shaded half cystines indicate cysteine residues involved in disulfide bonds.

**
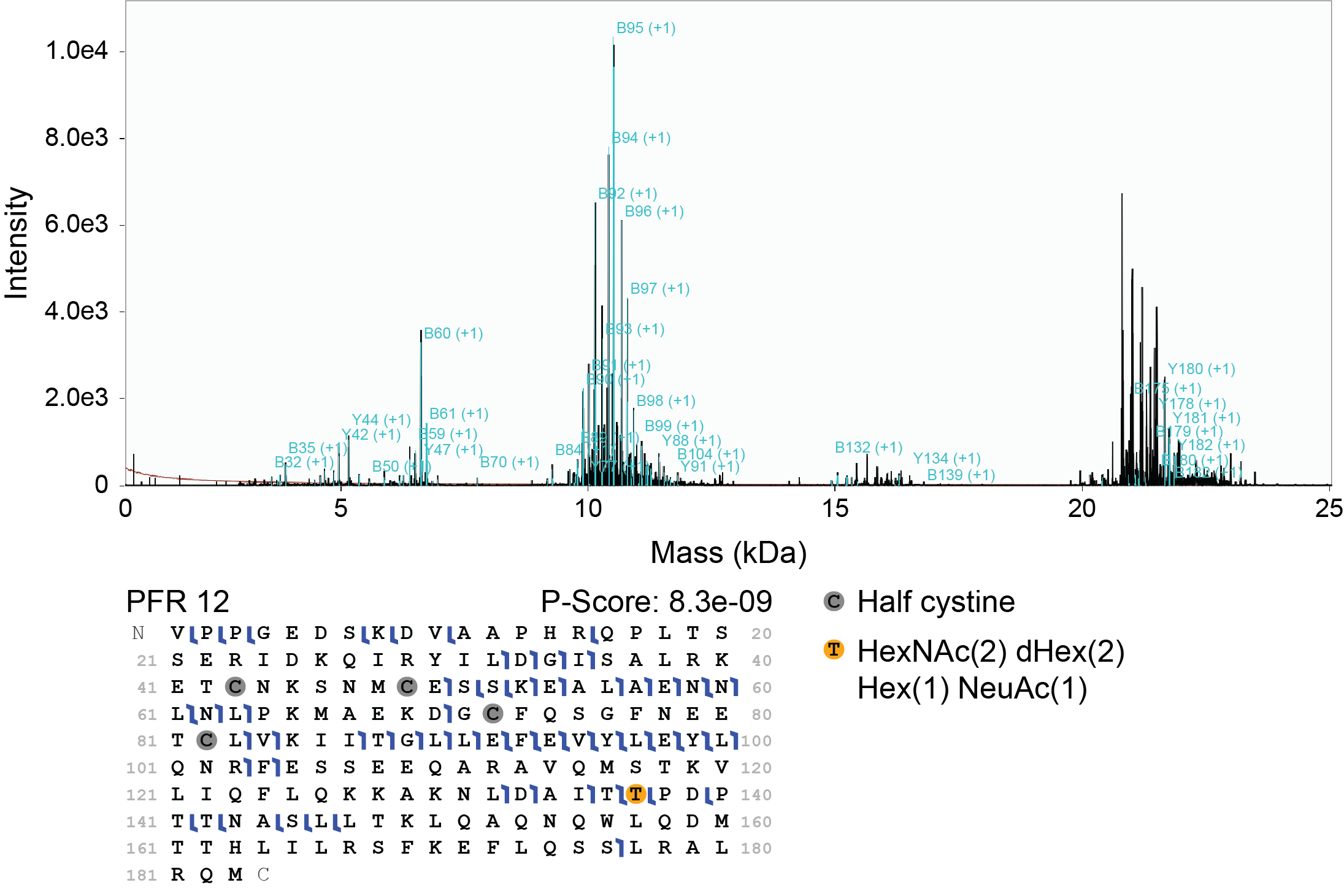
Fig. S9** I^2^MS^2^ spectrum and graphical fragment map for rhIL-6 proteoform 12. Shaded half cystines indicate cysteine residues involved in disulfide bonds.

**
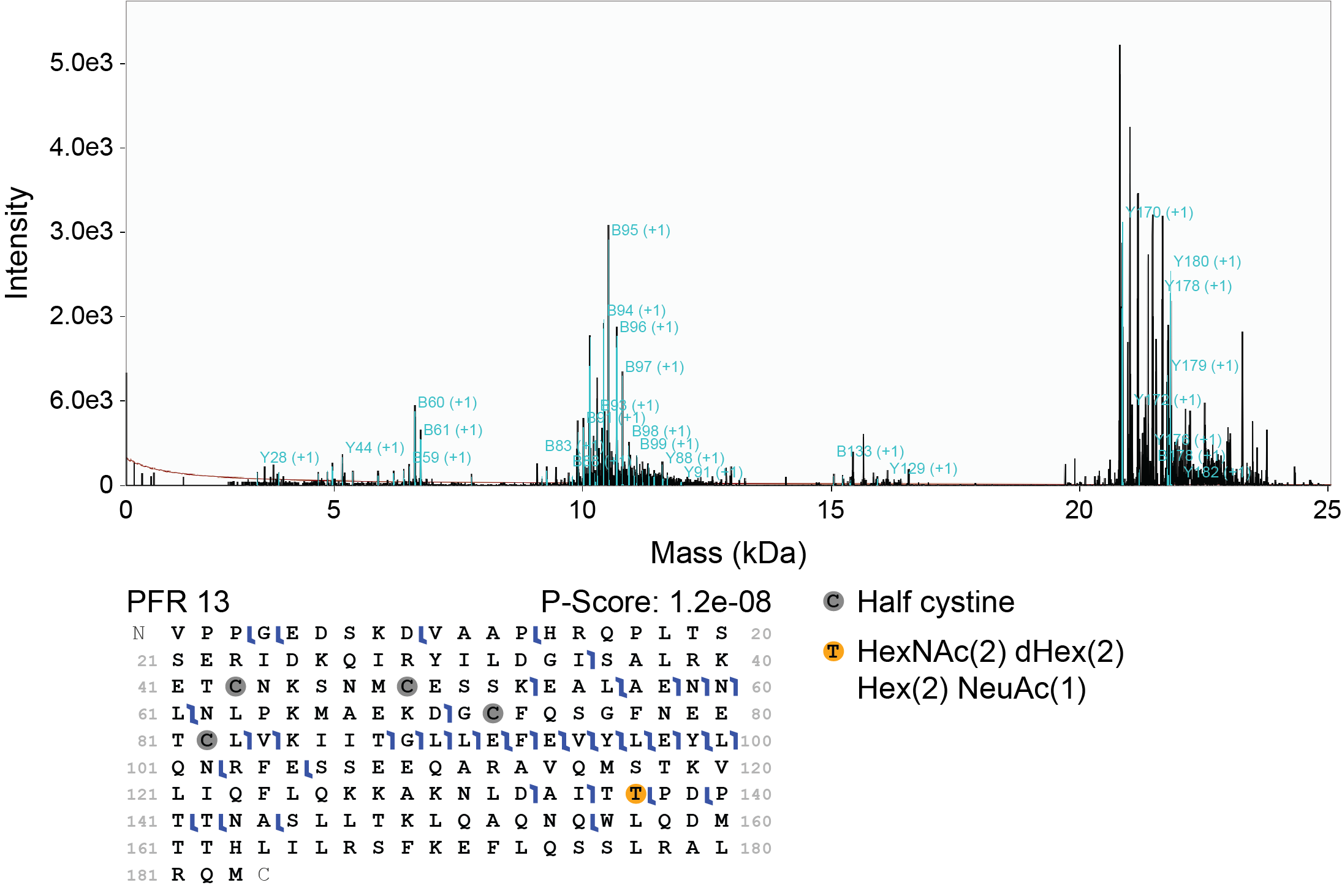
**

**Fig. S10** I^2^MS^2^ spectrum and graphical fragment map for rhIL-6 proteoform 13. Shaded half cystines indicate cysteine residues involved in disulfide bonds.
